# Bioconductor workflow for single-cell RNA sequencing: Normalization, dimensionality reduction, clustering, and lineage inference

**DOI:** 10.12688/f1000research.12122.1

**Published:** 2017-07-21

**Authors:** Fanny Perraudeau, Davide Risso, Kelly Street, Elizabeth Purdom, Sandrine Dudoit

**Affiliations:** 1 Graduate Group in Biostatistics, University of California, Berkeley, Berkeley, CA, 94720, USA; 2 Division of Biostatistics and Epidemiology, Department of Healthcare Policy and Research, Weill Cornell Medicine, New York, NY, 10065, USA; 3 Department of Statistics, University of California, Berkeley, Berkeley, CA, 94720, USA; 4 Division of Biostatistics, University of California, Berkeley, Berkeley, CA, 94720, USA

**Keywords:** single-cell, RNA-seq, normalization, dimensionality reduction, clustering, lineage inference, differential expression, workflow

## Abstract

Novel single-cell transcriptome sequencing assays allow researchers to measure gene expression levels at the resolution of single cells and offer the unprecendented opportunity to investigate at the molecular level fundamental biological questions, such as stem cell differentiation or the discovery and characterization of rare cell types. However, such assays raise challenging statistical and computational questions and require the development of novel methodology and software. Using stem cell differentiation in the mouse olfactory epithelium as a case study, this integrated workflow provides a step-by-step tutorial to the methodology and associated software for the following four main tasks: (1) dimensionality reduction accounting for zero inflation and over dispersion and adjusting for gene and cell-level covariates; (2) cell clustering using resampling-based sequential ensemble clustering; (3) inference of cell lineages and pseudotimes; and (4) differential expression analysis along lineages.

## Introduction

Single-cell RNA sequencing (scRNA-seq) is a powerful and promising class of high-throughput assays that enable researchers to measure genome-wide transcription levels at the resolution of single cells. To properly account for features specific to scRNA-seq, such as zero inflation and high levels of technical noise, several novel statistical methods have been developed to tackle questions that include normalization, dimensionality reduction, clustering, the inference of cell lineages and pseudotimes, and the identification of differentially expressed (DE) genes. While each individual method is useful on its own for addressing a specific question, there is an increasing need for workflows that integrate these tools to yield a seamless scRNA-seq data analysis pipeline. This is all the more true with novel sequencing technologies that allow an increasing number of cells to be sequenced in each run. For example, the Chromium Single Cell 3’ Solution was recently used to sequence and profile about 1.3 million cells from embryonic mouse brains.

scRNA-seq low-level analysis workflows have already been developed, with useful methods for quality control (QC), exploratory data analysis (EDA), pre-processing, normalization, and visualization. The workflow described in Lun *et al.* (2016) and the package scater ( McCarthy *et al.*, 2017) are such examples based on open-source R software packages from the Bioconductor Project ( Huber *et al.*, 2015). In these workflows, single-cell expression data are organized in objects of the SCESet class allowing integrated analysis. However, these workflows are mostly used to prepare the data for further downstream analysis and do not focus on steps such as cell clustering and lineage inference.

**Figure 1.**
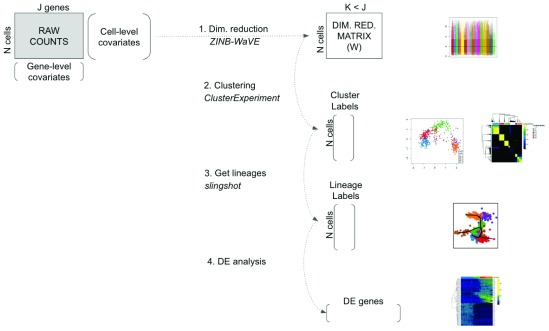
Workflow for analyzing scRNA-seq datasets. On the right, main plots generated by the workflow.

Here, we propose an integrated workflow for dowstream analysis, with the following four main steps: (1) dimensionality reduction accounting for zero inflation and over-dispersion, and adjusting for gene and cell-level covariates, using the zinbwave Bioconductor package; (2) robust and stable cell clustering using resampling-based sequential ensemble clustering, as implemented in the clusterExperiment Bioconductor package; (3) inference of cell lineages and ordering of the cells by developmental progression along lineages, using the slingshot R package; and (4) DE analysis along lineages. Throughout the workflow, we use a single SummarizedExperiment object to store the scRNA-seq data along with any gene or cell-level metadata available from the experiment See Figure 1.

## Analysis of olfactory stem cell differentiation using scRNA-seq data

### Overview

This workflow is illustrated using data from a scRNA-seq study of stem cell differentiation in the mouse olfactory epithelium (OE) ( Fletcher *et al.*, 2017). The olfactory epithelium contains mature olfactory sensory neurons (mOSN) that are continuously renewed in the epithelium via neurogenesis through the differentiation of globose basal cells (GBC), which are the actively proliferating cells in the epithelium. When a severe injury to the entire tissue happens, the olfactory epithelium can regenerate from normally quiescent stem cells called horizontal basal cells (HBC), which become activated to differentiate and reconstitute all major cell types in the epithelium.

The scRNA-seq dataset we use as a case study was generated to study the differentiation of HBC stem cells into different cell types present in the olfactory epithelium. To map the developmental trajectories of the multiple cell lineages arising from HBCs, scRNA-seq was performed on FACS-purified cells using the Fluidigm C1 microfluidics cell capture platform followed by Illumina sequencing. The expression level of each gene in a given cell was quantified by counting the total number of reads mapping to it. Cells were then assigned to different lineages using a statistical analysis pipeline analogous to that in the present workflow. Finally, results were validated experimentally using *in vivo* lineage tracing. Details on data generation and statistical methods are available in Fletcher *et al.* (2017); Risso *et al.* (2017); Street *et al.* (2017).

**Figure 2.**
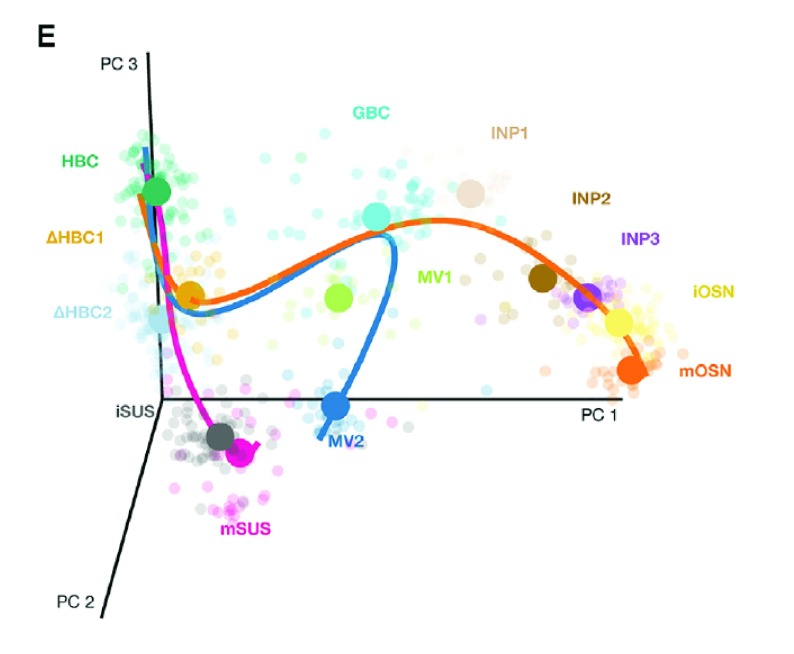
Stem cell differentiation in the mouse olfactory epithelium. Reprinted from Cell Stem Cell, Vol 20, Fletcher *et al.*, Deconstructing Olfactory Stem Cell Trajectories at Single-Cell Resolution Pages No. 817–830, Copyright (2017), with permission from Elsevier.

It was found that the first major bifurcation in the HBC lineage trajectory occurs prior to cell division, producing either mature sustentacular (mSUS) cells or GBCs. Then, the GBC lineage, in turn, branches off to give rise to mOSN and microvillous (MV) ( Figure 2). In this workflow, we describe a sequence of steps to recover the lineages found in the original study, starting from the genes by cells matrix of raw counts publicly available on the NCBI Gene Expression Omnibus with accession GSE95601.

### Package versions

The following packages are needed.



                        # Bioconductor

                        library
                        (BiocParallel)

                        library
                        (clusterExperiment)

                        library
                        (scone)

                        library
                        (zinbwave)


                        # GitHub

                        library
                        (slingshot)


                        # CRAN

                        library
                        (doParallel)

                        library
                        (gam)

                        library
                        (RColorBrewer)


                        set.seed
                        (
                        20
                        )
                    


Note that in order to successfully run the workflow, we need the following versions of the Bioconductor packages scone (1.1.2), zinbwave (0.99.6), and clusterExperiment (1.3.2). We recommend running Bioconductor 3.6 (currently the devel version; see https://www.bioconductor.org/developers/how-to/useDevel/).

### Parallel computation

To give the user an idea of the time needed to run the workflow, the function system.time was used to report computation times for the time-consuming functions. Computations were performed with 2 cores on a MacBook Pro (early 2015) with a 2.7 GHz Intel Core i5 processor and 8 GB of RAM. The Bioconductor package iocParallel was used to allow for parallel computing in the zinbwave function. Users with a different operating system may change the package used for parallel computing and the NCORES variable below.



                        NCORES <- 
                        2

                        mysystem = 
                        Sys.info
                        ()[[
                        "sysname"
                        ]]

                        if (mysystem == 
                        "Darwin"
                        ){
  
                        registerDoParallel
                        (NCORES)
  
                        register
                        (
                        DoparParam
                        ())

                        }else if (mysystem == 
                        "Linux"
                        ){
  
                        register
                        (
                        bpstart
                        (
                        MulticoreParam
                        (
                        workers=
                        NCORES)))

                        }else{
  
                        print
                        (
                        "Please change this to allow parallel computing on your computer."
                        )
  
                        register
                        (
                        SerialParam
                        ())

                        }
                    


### Pre-processing

Counts for all genes in each cell were obtained from NCBI Gene Expression Omnibus (GEO), with accession number GSE95601. Before filtering, the dataset had 849 cells and 28,361 detected genes (i.e., genes with non-zero read counts).

Note that in the following, we assume that the user has access to a data folder located at ../data. Users with a different directory structure may need to change the data_dir variable below to reproduce the workflow.



                        data_dir <- 
                        "../data/"


                        urls = 
                        c
                        (
                        "https://www.ncbi.nlm.nih.gov/geo/download/?acc=GSE95601&format=file&file=GSE95601%5FoeHBCdiff%
          
                        "https://raw.githubusercontent.com/rufletch/p63-HBC-diff/master/ref/oeHBCdiff_clusterLabels.
                        )


                        if(!
                        file.exists
                        (
                        paste0
                        (data_dir, 
                        "GSE95601_oeHBCdiff_Cufflinks_eSet.Rda"
                        ))) {
  
                        download.file
                        (urls[
                        1
                        ], 
                        paste0
                        (data_dir, 
                        "GSE95601_oeHBCdiff_Cufflinks_eSet.Rda.gz"
                        ))
  
                        R.utils::
                        gunzip
                        (
                        paste0
                        (data_dir, 
                        "GSE95601_oeHBCdiff_Cufflinks_eSet.Rda.gz"
                        ))

                        }


                        if(!
                        file.exists
                        (
                        paste0
                        (data_dir, 
                        "oeHBCdiff_clusterLabels.txt"
                        ))) {
  
                        download.file
                        (urls[
                        2
                        ], 
                        paste0
                        (data_dir, 
                        "oeHBCdiff_clusterLabels.txt"
                        ))

                        }
                    




                        load
                        (
                        paste0
                        (data_dir, 
                        "GSE95601_oeHBCdiff_Cufflinks_eSet.Rda"
                        ))


                        # Count matrix

                        E <- 
                        assayData
                        (Cufflinks_eSet)$counts_table


                        # Remove undetected genes

                        E <- 
                        na.omit
                        (E)

                        E <- E[
                        rowSums
                        (E)>
                        0
                        ,]

                        dim
                        (E)


                        ## [1] 28361 849
                    


We remove the ERCC spike-in sequences and the CreER gene, as the latter corresponds to the estrogen receptor fused to Cre recombinase (Cre-ER), which is used to activate HBCs into differentiation following injection of tamoxifen (see Fletcher *et al.* (2017) for details).



                        # Remove ERCC and CreER genes

                        cre <- E[
                        "CreER"
                        ,]

                        ercc <- E[
                        grep
                        (
                        "^ERCC-"
                        , 
                        rownames
                        (E)),]

                        E <- E[
                        grep
                        (
                        "^ERCC-"
                        , 
                        rownames
                        (E), 
                        invert = 
                        TRUE
                        ), ]

                        E <- E[-
                        which
                        (
                        rownames
                        (E)==
                        "CreER"
                        ), ]

                        dim
                        (E)


                        ## [1] 28284  849
                    


Throughout the workflow, we use the class SummarizedExperiment to keep track of the counts and their associated metadata within a single object. The cell-level metadata contain quality control measures, sequencing batch ID, and cluster and lineage labels from the original publication ( Fletcher *et al.*, 2017). Cells with a cluster label of -2 were not assigned to any cluster in the original publication.



                        # Extract QC metrics

                        qc <- 
                        as.matrix
                        (
                        protocolData
                        (Cufflinks_eSet)@data)[,
                        c
                        (
                        1
                        :
                        5
                        , 
                        10
                        :
                        18
                        )]

                        qc <- 
                        cbind
                        (qc
                        , 
                        CreER = 
                        cre
                        , 
                        ERCC_reads = colSums
                        (ercc))


                        # Extract metadata

                        batch <- 
                        droplevels
                        (
                        pData
                        (Cufflinks_eSet)$MD_c1_run_id)

                        bio <- 
                        droplevels
                        (
                        pData
                        (Cufflinks_eSet)$MD_expt_condition)

                        clusterLabels <- 
                        read.table
                        (
                        paste0
                        (data_dir, 
                        "oeHBCdiff_clusterLabels.txt"
                        ),
                               
                        sep = 
                        "\t", 
                        stringsAsFactors = 
                        FALSE
                        )

                        m <- 
                        match
                        (
                        colnames
                        (E), clusterLabels[, 
                        1
                        ])


                        # Create metadata data.frame

                        metadata <- 
                        data.frame
                        (
                        "Experiment" 
                        = bio,
                          
                        "Batch" 
                        = batch,
                          
                        "publishedClusters" 
                        = clusterLabels[m,
                        2
                        ],
                       qc)


                        # Symbol for cells not assigned to a lineage in original data

                        metadata$publishedClusters[
                        is.na
                        (metadata$publishedClusters)] <- -
                        2


                        se <- 
                        SummarizedExperiment
                        (
                        assays = list
                        (
                        counts = 
                        E),
                              
                        colData = 
                        metadata)

                        se
                    




                        ## class: SummarizedExperiment
## dim: 28284 849
## metadata(0):
## assays(1): counts
## rownames(28284): Xkr4 LOC102640625 ... Ggcx.1 eGFP
## rowData names(0):
## colnames(849): OEP01_N706_S501 OEP01_N701_S501 ... OEL23_N704_S503
##   OEL23_N703_S502
## colData names(19): Experiment Batch ... CreER ERCC_reads
                    


**Figure 3.**
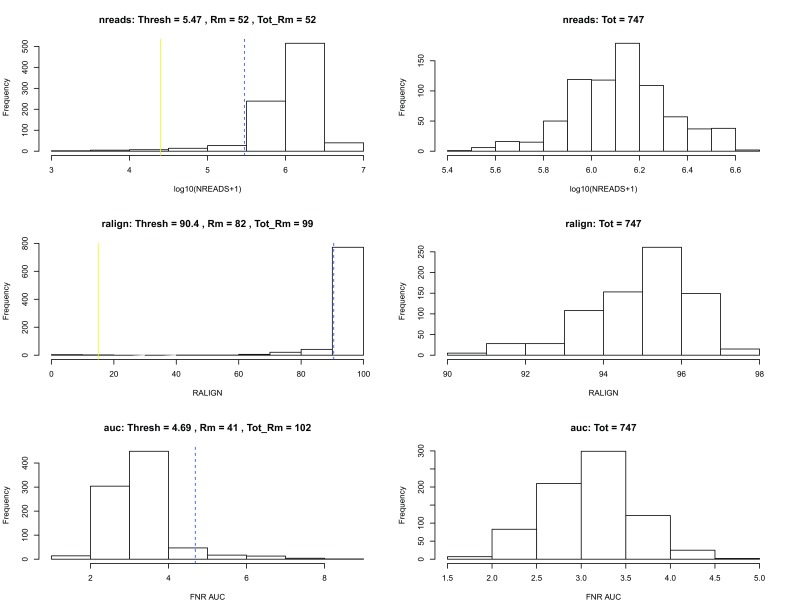
SCONE: Filtering of low-quality cells.

Using the Bioconductor R package scone, we remove low-quality cells according to the quality control filter implemented in the function metric_sample_filter and based on the following criteria ( Figure 3): (1) Filter out samples with low total number of reads or low alignment percentage and (2) filter out samples with a low detection rate for housekeeping genes. See the scone vignette for details on the filtering procedure.



                        # QC-metric-based sample-filtering

                        data
                        (
                        "housekeeping"
                        )

                        hk = 
                        rownames
                        (se)[
                        toupper
                        (
                        rownames
                        (se)) %in% housekeeping$V1]


                        mfilt <- 
                        metric_sample_filter
                        (
                        assay
                        (se),
                                  
                        nreads = colData
                        (se)$NREADS,
                                  
                        ralign = colData
                        (se)$RALIGN,
                                  
                        pos_controls = rownames
                        (se) %in% hk,
                                  
                        zcut = 
                        3
                        , 
                        mixture = 
                        FALSE
                        ,
                                  
                        plot = 
                        TRUE)


After sample filtering, we are left with 747 good quality cells.



                        # Simplify to a single logical

                        mfilt <- !
                        apply
                        (
                        simplify2array
                        (mfilt[!
                        is.na
                        (mfilt)]), 
                        1
                        , any)

                        se <- se[, mfilt]

                        dim
                        (se)
                    




                        ## [1] 28284 747
                    


Finally, for computational efficiency, we retain only the 1,000 most variable genes. This seems to be a reasonnable choice for the illustrative purpose of this workflow, as we are able to recover the biological signal found in the published analysis ( Fletcher *et al.*, 2017). In general, however, we recommend care in selecting a gene filtering scheme, as an appropriate choice is dataset-dependent.



                        # Filtering to top 1,000 most variable genes

                        vars <- 
                        rowVars
                        (
                        log1p(
                        
                            assay
                        
                        (se)))

                        names
                        (vars) <- 
                        rownames
                        (se)

                        vars <- 
                        sort
                        (vars, 
                        decreasing = 
                        TRUE
                        )

                        core <- se[
                        names
                        (vars)[
                        1
                        :
                        1000
                        ],]
                    


### Dataset structure

Overall, after the above pre-processing steps, our dataset has 1,000 genes and 747 cells.



                        core

## class: SummarizedExperiment
## dim: 1000 747
## metadata(0):
## assays(1): counts
## rownames(1000): Cbr2 Cyp2f2 ... Rnf13 Atp7b
## rowData names(0):
## colnames(747): OEP01_N706_S501 OEP01_N701_S501 ... OEL23_N704_S503
##   OEL23_N703_S502
## colData names(19): Experiment Batch ... CreER ERCC_reads
                    


Metadata for the cells are stored in the slot colData from the SummarizedExperiment object. Cells were processed in 18 different batches.



                        batch <- 
                        colData
                        (core)$Batch

                        col_batch = 
                        c
                        (
                        brewer.pal
                        (
                        9
                        , 
                        "Set1"
                        ), 
                        brewer.pal
                        (
                        8
                        , 
                        "Dark2"),
                
                        brewer.pal(
                        8, 
                        "Accent"
                        )[
                        1
                        ])

                        names
                        (col_batch) = 
                        unique
                        (batch)

                        table
                        (batch)
                    




                        ## batch
## GBC08A GBC08B GBC09A GBC09B P01 P02 P03A P03B P04 P05
##     39     40     35     22  31  48   51   40  20  23
##    P06    P10    P11    P12 P13 P14  Y01  Y04
##     51     40     50     50  60  47   58   42
                    


In the original work ( Fletcher *et al.*, 2017), cells were clustered into 14 different clusters, with 151 cells not assigned to any cluster (i.e., cluster label of -2).



                        publishedClusters <- 
                        colData
                        (core)[, 
                        "publishedClusters"
                        ]

                        col_clus <- 
                        c
                        (
                        "transparent"
                        , 
                        "#1B9E77"
                        , 
                        "antiquewhite2"
                        , 
                        "cyan"
                        , 
                        "#E7298A"
                        ,
                
                        "#A6CEE3"
                        , 
                        "#666666"
                        , 
                        "#E6AB02"
                        , 
                        "#FFED6F"
                        , 
                        "darkorchid2"
                        ,
                
                        "#B3DE69"
                        , 
                        "#FF7F00"
                        , 
                        "#A6761D"
                        , 
                        "#1F78B4"
                        )

                        names
                        (col_clus) <- 
                        sort
                        (
                        unique
                        (publishedClusters))

                        table
                        (publishedClusters)


                        ## publishedClusters
##  -2   1   2   3   4   5   7   8   9  10  11  12  14  15
## 151  90  25  54  35  93  58  27  74  26  21  35  26  32
                    


Note that there is partial nesting of batches within clusters (i.e., cell type), which could be problematic when correcting for batch effects in the dimensionality reduction step below.



                        table
                        (
                        data.frame
                        (
                        batch = as.vector
                        (batch),
                   
                        cluster = 
                        publishedClusters))


                        ##         cluster
## batch    -2  1  2  3  4  5  7  8  9 10 11 12 14 15
##   GBC08A  3  0  2 12  9  0  0  0  0  0  2  0  2  9
##   GBC08B  8  0  7  5  3  0  0  0  1  2  3  0  5  6
##   GBC09A  6  0  1  5  8  0  0  0  1  1  0  0  6  7
##   GBC09B 12  0  2  1  3  0  0  0  1  0  0  0  3  0
##   P01     7  0  2  4  3 15  0  0  0  0  0  0  0  0
##   P02     5  2  0  9  3 15  3  3  2  3  0  2  1  0
##   P03A   15  3  0  2  0 12  2  9  4  2  0  2  0  0
##   P03B    9  1  2  1  1 11  1  2  8  1  1  2  0  0
##   P04     8  0  0  0  0  9  1  0  1  1  0  0  0  0
##   P05     3  0  0  0  1 11  3  0  1  0  2  2  0  0
##   P06    12  1  2  3  0  8  2  4  8  4  1  2  2  2
##   P10     7  3  1  4  0  3  5  8  1  0  2  5  0  1
##   P11     6  2  1  1  0  1  5  1 22  3  1  6  0  1
##   P12    10  0  2  0  0  4 10  0  8  2  3  6  4  1
##   P13    13  1  2  4  0  4 15  0  4  5  6  1  3  2
##   P14     9  0  0  1  2  0 11  0 12  2  0  7  0  3
##   Y01     8 46  1  1  2  0  0  0  0  0  0  0  0  0
##   Y04    10 31  0  1  0  0  0  0  0  0  0  0  0  0
                    


### Normalization and dimensionality reduction: ZINB-WaVE

In scRNA-seq analysis, dimensionality reduction is often used as a preliminary step prior to downstream analyses, such as clustering, cell lineage and pseudotime ordering, and the identification of DE genes. This allows the data to become more tractable, both from a statistical (cf. curse of dimensionality) and computational point of view. Additionally, technical noise can be reduced while preserving the often intrinsically low-dimensional signal of interest ( Dijk *et al.*, 2017; Pierson & Yau, 2015; Risso *et al.*, 2017).

**Figure 4.**
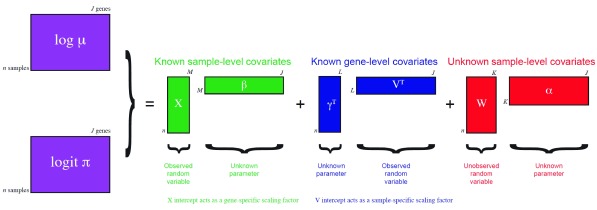
ZINB-WaVE: Schematic view of the ZINB-WaVE model. This figure was reproduced with kind permission from Risso *et al.* (2017).

Here, we perform dimensionality reduction using the zero-inflated negative binomial-based wanted variation extraction (ZINB-WaVE) method implemented in the Bioconductor R package zinbwave. The method fits a ZINB model that accounts for zero inflation (dropouts), over-dispersion, and the count nature of the data. The model can include a cell-level intercept, which serves as a global-scaling normalization factor. The user can also specify both gene-level and cell-level covariates. The inclusion of observed and unobserved cell-level covariates enables normalization for complex, non-linear effects (often referred to as batch effects), while gene-level covariates may be used to adjust for sequence composition effects (e.g., gene length and GC-content effects). A schematic view of the ZINB-WaVE model is provided in Figure 4. For greater detail about the ZINB-WaVE model and estimation procedure, please refer to the original manuscript ( Risso *et al.*, 2017).

As with most dimensionality reduction methods, the user needs to specify the number of dimensions for the new low-dimensional space. Here, we use K = 50 dimensions and adjust for batch effects via the matrix X.

Note that if the users include more genes in the analysis, it may be preferable to reduce K to achieve a similar computational time.



                        print
                        (
                        system.time
                        (se <- 
                        zinbwave
                        (core, 
                        K = 
                        50
                        , 
                        X = 
                        "~ Batch"
                        ,
                                     
                        residuals = 
                        TRUE
                        ,
                                     
                        normalizedValues = 
                        TRUE
                        )))



                        ##     user  system  elapsed
## 3262.127 678.170 2154.357
                    


### Normalization

The function zinbwave returns a SummarizedExperiment object that includes normalized expression measures, defined as deviance residuals from the fit of the ZINB-WaVE model with user-specified gene- and cell-level covariates. Such residuals can be used for visualization purposes (e.g., in heatmaps, boxplots). Note that, in this case, the low-dimensional matrix W is not included in the computation of residuals to avoid the removal of the biological signal of interest.



                        norm <- 
                        assays
                        (se)$normalizedValues

                        norm[
                        1
                        :
                        3
                        ,
                        1
                        :
                        3
                        ]



                        ##       OEP01_N706_S501 OEP01_N701_S501 OEP01_N707_S507
## Cbr2         4.557371        4.375069       -4.142697
## Cyp2f2       4.321644        4.283266        4.090283
## Gstm1        4.796498        4.663366        4.416324
                    


**Figure 5.**
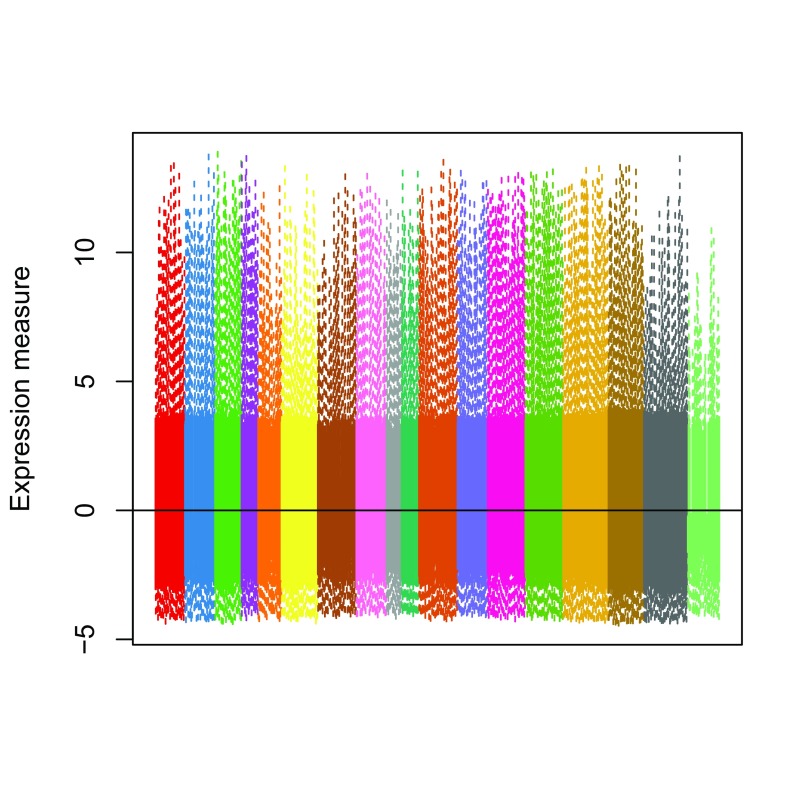
ZINB-WaVE: Boxplots of normalized expression measures (deviance residuals), color-coded by batch.



As expected, the normalized values no longer exhibit batch effects ( Figure 5).

                        norm_order <- norm[, 
                        order
                        (
                        as.numeric
                        (batch))]

                        col_order <- col_batch[batch[
                        order
                        (
                        as.numeric
                        (batch))]]

                        boxplot
                        (norm_order, 
                        col = 
                        col_order, 
                        staplewex = 
                        0, 
                        outline = 
                        0,
         
                        border = 
                        col_order, 
                        xaxt = 
                        "n"
                        , 
                        ylab=
                        "Expression measure"
                        )

                        abline
                        (
                        h=
                        0
                        )
                    


**Figure 6.**
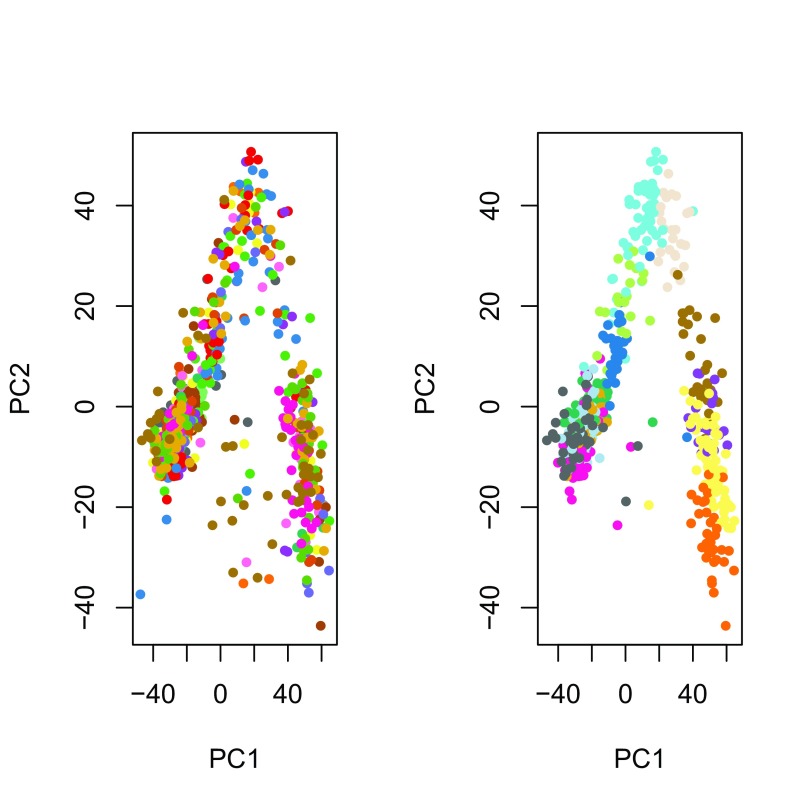
ZINB-WaVE: PCA of normalized expression measures, where each point represents a cell. Cells are color-coded by batch (left panel) and by original published clustering (right panel).



The principal component analysis (PCA) of the normalized values shows that, as expected, cells do not cluster by batch, but by the original clusters ( Figure 6). Overall, it seems that normalization was effective at removing batch effects without removing biological signal, in spite of the partial nesting of batches within clusters.

                        pca <- 
                        prcomp
                        (
                        t
                        (norm))

                        par
                        (
                        mfrow = c
                        (
                        1,
                        2
                        ))

                        plot
                        (pca$x, 
                        col = 
                        col_batch[batch], 
                        pch = 
                        20
                        , 
                        main = 
                        ""
                        )

                        plot
                        (pca$x, 
                        col = 
                        col_clus[
                        as.character
                        (publishedClusters)], 
                        pch = 
                        20
                        , 
                        main = 
                        ""
                        )
                    


### Dimensionality reduction

**Figure 7.**
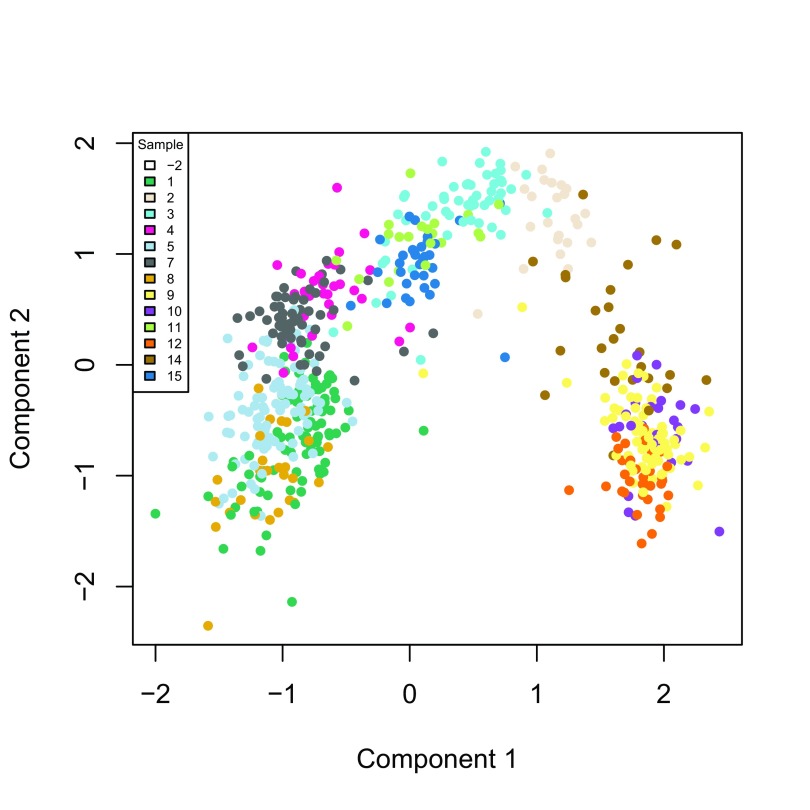
ZINB-WaVE: MDS of the low-dimensional matrix W, where each point represents a cell and cells are color-coded by original published clustering.



The zinbwave function can also be used to perform dimensionality reduction, where, in this workflow, the user-supplied dimension K of the low-dimensional space is set to K = 50. The resulting low-dimensional matrix W can be visualized in two dimensions by performing multi-dimensional scaling (MDS) using the Euclidian distance. To verify that W indeed captures the biological signal of interest, we display the MDS results in a scatterplot with colors corresponding to the original published clusters ( Figure 7).

                        W <- 
                        colData
                        (se)[, 
                        grepl
                        (
                        "^W"
                        , 
                        colnames
                        (
                        colData
                        (se)))]

                        W <- 
                        as.matrix
                        (W)

                        d <- 
                        dist
                        (W)

                        fit <- 
                        cmdscale
                        (d, 
                        eig = 
                        TRUE
                        , 
                        k = 
                        2
                        )

                        plot
                        (fit$points, 
                        col = 
                        col_clus[
                        as.character
                        (publishedClusters)], 
                        main = 
                        ""
                        ,
      
                        pch = 
                        20
                        , 
                        xlab = 
                        "Component 1"
                        , 
                        ylab = 
                        "Component 2"
                        )

                        legend
                        (
                        x = 
                        "topleft"
                        , 
                        legend = unique
                        (
                        names
                        (col_clus)), 
                        cex = 
                        .
                        5
                        ,
        
                        fill = unique
                        (col_clus), 
                        title = 
                        "Sample"
                        )
                    


### Cell clustering: RSEC

The next step of the workflow is to cluster the cells according to the low-dimensional matrix W computed in the previous step. We use the resampling-based sequential ensemble clustering (RSEC) framework implemented in the RSEC function from the Bioconductor R package clusterExperiment. Specifically, given a set of user-supplied base clustering algorithms and associated tuning parameters (e.g., *k*-means, with a range of values for *k*), RSEC generates a collection of candidate clusterings, with the option of resampling cells and using a sequential tight clustering procedure, as in Tseng & Wong (2005). A consensus clustering is obtained based on the levels of co-clustering of samples across the candidate clusterings. The consensus clustering is further condensed by merging similar clusters, which is done by creating a hierarchy of clusters, working up the tree, and testing for differential expression between sister nodes, with nodes of insufficient DE collapsed. As in supervised learning, resampling greatly improves the stability of clusters and considering an ensemble of methods and tuning parameters allows us to capitalize on the different strengths of the base algorithms and avoid the subjective selection of tuning parameters.

Note that the defaults in RSEC are designed for input data that are the actual (normalized) counts. Here, we are applying RSEC instead to the low-dimensional W matrix from ZINB-WaVE, for which we make a separate SummarizedExperiment object. For this reason, we choose to not use certain options in RSEC. In particular, we do not use the default dimensionality reduction step, since our input W is already in a space of reduced dimension. Specifically, RSEC offers a dimensionality reduction option for the input to both the clustering routines ( dimReduce) and the construction of the hiearchy between the clusters ( dendroReduce). We also skip the option to merge our clusters based on the amount of differential gene expression between clusters.



                        seObj <- 
                        SummarizedExperiment
                        (
                        t
                        (W), 
                        colData = colData
                        (core))

                        print
                        (
                        system.time
                        (ceObj <- 
                        RSEC
                        (seObj, 
                        k0s = 
                        4
                        :
                        15
                        , 
                        alphas = c
                        (
                        0.1
                        ),
                                    
                        betas = 
                        0.8
                        , 
                        dimReduce=
                        "none"
                        ,
                                    
                        clusterFunction = 
                        "hierarchical01"
                        , 
                        minSizes=
                        1
                        ,
                                    
                        ncores = 
                        NCORES, 
                        isCount=
                        FALSE
                        ,
                                    
                        dendroReduce=
                        "none"
                        ,
                        dendroNDims=
                        NA
                        ,
                                    
                        subsampleArgs = list
                        (
                        resamp.num=
                        
                            100
                        
                        ,
                                                            
                        clusterFunction=
                        "kmeans"
                        ,
                                                            
                        clusterArgs=list
                        (
                        nstart=
                        10
                        )),
                                    
                        verbose=
                        TRUE
                        ,
                                    
                        combineProportion = 
                        0.7
                        ,
                                    
                        mergeMethod = 
                        "none"
                        , 
                        random.seed=
                        424242
                        ,
                                    
                        combineMinSize = 
                        10
                        )))
                    




                        ## Note: clusters will not be merged because argument ’mergeMethod’ was not given (or was equal to ’
##     user   system  elapsed
## 4083.942  187.405 5069.999
                    


**Figure 8.**
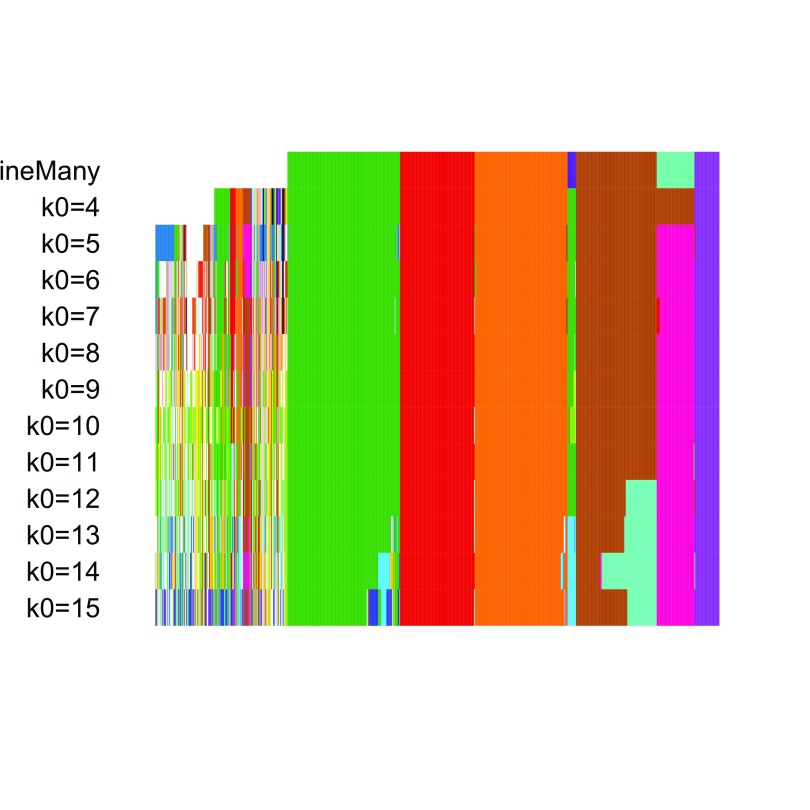
RSEC: Candidate clusterings found using the function RSEC from the clusterExperiment package.



The resulting candidate clusterings can be visualized using the plotClusters function ( Figure 8), where columns correspond to cells and rows to different clusterings. Each sample is color-coded based on its clustering for that row, where the colors have been chosen to try to match up clusters that show large overlap accross rows. The first row correspond to a consensus clustering across all candidate clusterings.

                        plotClusters
                        (ceObj, 
                        colPalette = c
                        (bigPalette, 
                        rainbow
                        (
                        199
                        )))
                    


**Figure 9.**
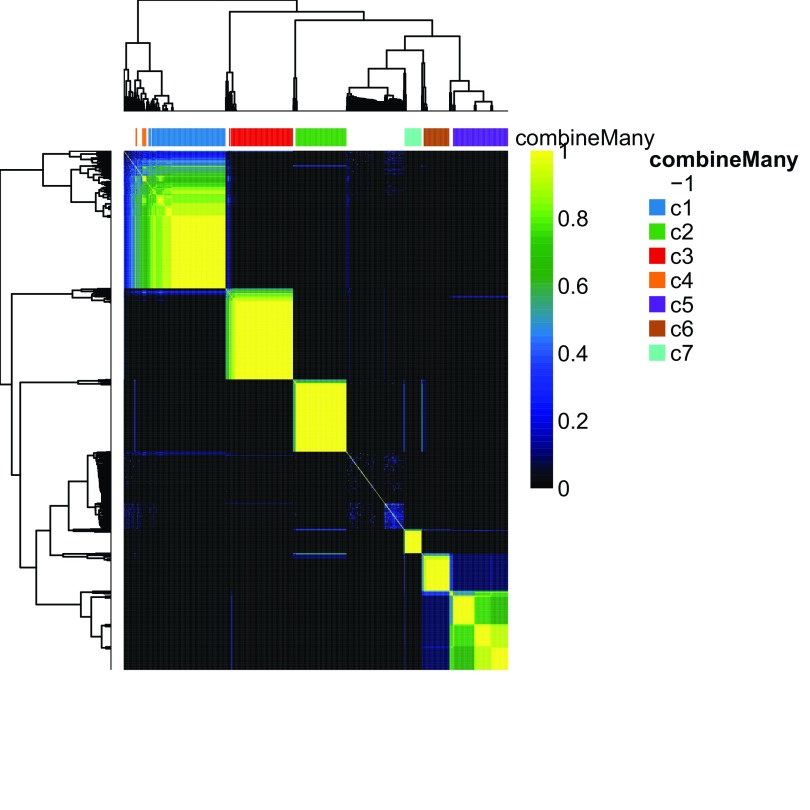
RSEC: Heatmap of co-clustering matrix.



The plotCoClustering function produces a heatmap of the co-clustering matrix, which records, for each pair of cells, the proportion of times they were clustered together across the candidate clusterings ( Figure 9).

                        plotCoClustering
                        (ceObj)
                    


**Figure 10.**
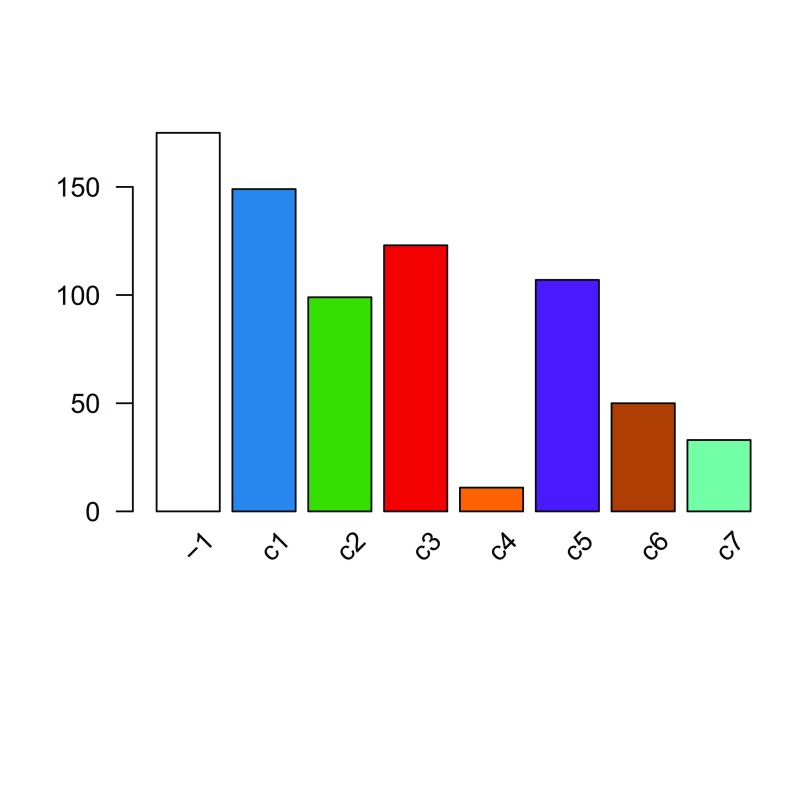
RSEC: Barplot of number of cells per cluster for our workflow’s RSEC clustering.



The distribution of cells across the consensus clusters can be visualized in Figure 10 and is as follows:

                        table
                        (
                        primaryClusterNamed
                        (ceObj))


                        ##
##  -1  c1  c2  c3  c4  c5  c6  c7
## 175 149  99 123  11 107  50  33


                        plotBarplot
                        (ceObj, 
                        legend = 
                        FALSE
                        )
                    


**Figure 11.**
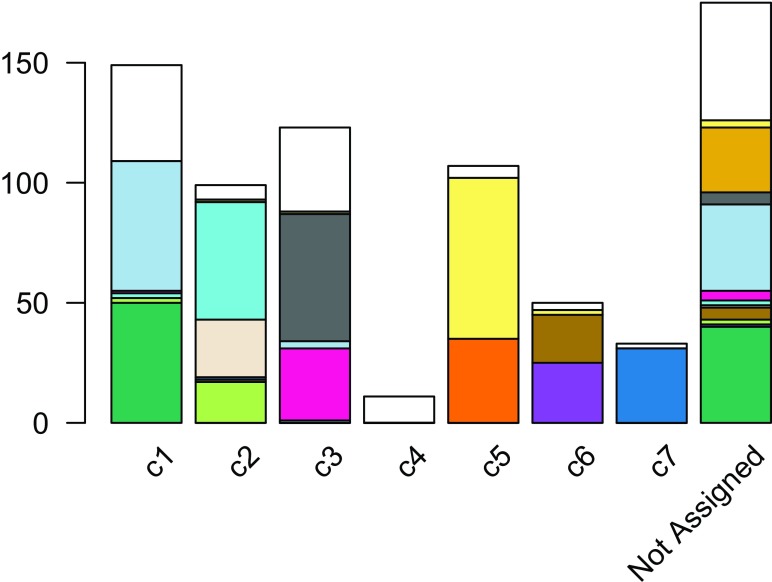
RSEC: Barplot of number of cells per cluster, for our workflow’s RSEC clustering, color-coded by original published clustering.



The distribution of cells in our workflow’s clustering overall agrees with that in the original published clustering ( Figure 11), the main difference being that several of the published clusters were merged here into single clusters. This discrepancy is likely caused by the fact that we started with the top 1,000 genes, which might not be enough to discriminate between closely related clusters.

                        ceObj <- 
                        addClusters
                        (ceObj, 
                        colData
                        (ceObj)$publishedClusters,
		        
                        clusterLabel = 
                        "publishedClusters"
                        )


                        ## change default color to match with Figure 7

                        clusterLegend
                        (ceObj)$publishedClusters[, 
                        "color"
                        ] <-
  
                        col_clus[
                        clusterLegend
                        (ceObj)$publishedClusters[, 
                        "name"
                        ]]


                        plotBarplot
                        (ceObj, 
                        whichClusters=c
                        (
                        "combineMany"
                        ,
                        "publishedClusters"
                        ),
	      
                        xlab = 
                        ""
                        , 
                        legend = 
                        FALSE
                        )
                    


**Figure 12.**
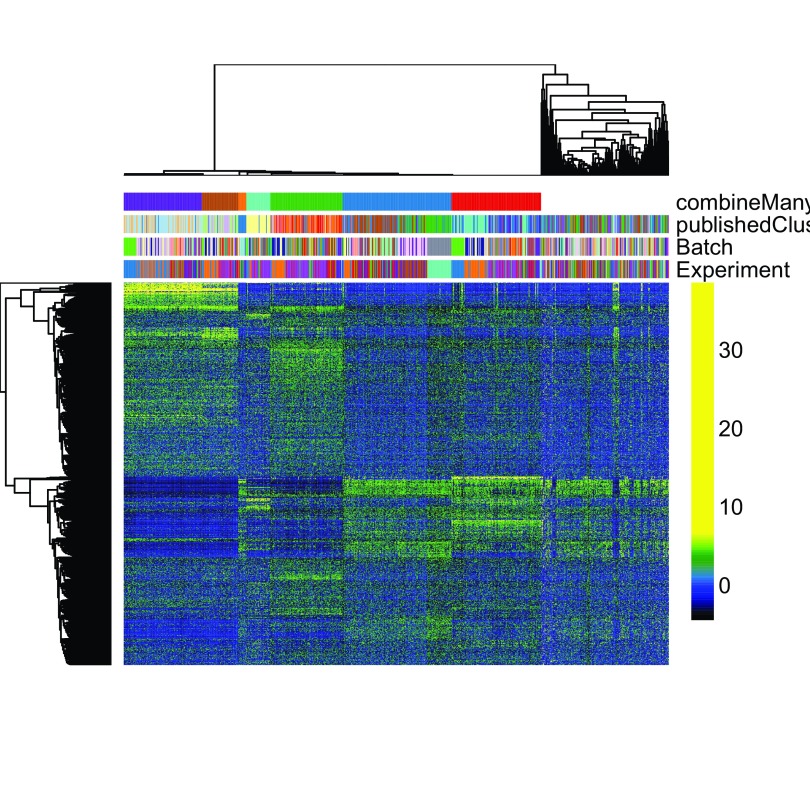
RSEC: Heatmap of the normalized expression measures for the 1,000 most variable genes, where rows correspond to genes and columns to cells ordered by RSEC clusters.



Figure 12 displays a heatmap of the normalized expression measures for the 1,000 most variable genes, where cells are clustered according to the RSEC consensus.

                        # Set colors for cell clusterings

                        colData
                        (ceObj)$publishedClusters <- 
                        as.factor
                        (
                        colData
                        (ceObj)$publishedClusters)

                        origClusterColors <- bigPalette[
                        1
                        :
                        nlevels
                        (
                        colData
                        (ceObj)$publishedClusters)]

                        experimentColors <- bigPalette[
                        1
                        :
                        nlevels
                        (
                        colData
                        (ceObj)$Experiment)]

                        batchColors <- bigPalette[
                        1
                        :
                        nlevels
                        (
                        colData
                        (ceObj)$Batch)]

                        metaColors <- 
                        list
                        (
                        "Experiment" 
                        = experimentColors,
                     
                        "Batch" 
                        = batchColors,
                     
                        "publishedClusters" 
                        = origClusterColors)


                        plotHeatmap
                        (ceObj, 
                        visualizeData = assays
                        (se)$normalizedValues,
              
                        whichClusters = 
                        "primary"
                        , 
                        clusterFeaturesData = 
                        "all"
                        ,
              
                        clusterSamplesData = 
                        "dendrogramValue"
                        , 
                        breaks = 
                        0.99
                        ,
              
                        sampleData = c
                        (
                        "publishedClusters"
                        , 
                        "Batch"
                        , 
                        "Experiment"
                        ),
              
                        clusterLegend = 
                        metaColors, 
                        annLegend = 
                        FALSE
                        , 
                        main = 
                        ""
                        )
                    


**Figure 13.**
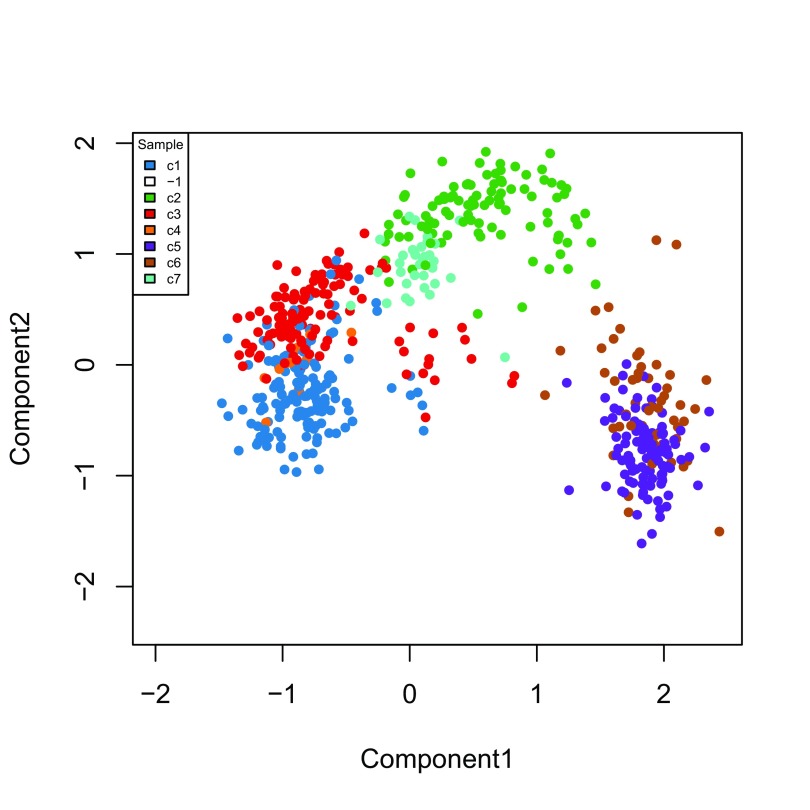
RSEC: MDS of the low-dimensional matrix W, where each point represents a cell and cells are color-coded by RSEC clustering.



Finally, we can visualize the cells in a two-dimensional space using the MDS of the low-dimensional matrix W and coloring the cells according to their newly-found RSEC clusters ( Figure 13); this is anologous to Figure 7 for the original published clusters.

                        palDF <- ceObj@clusterLegend[[
                        1
                        ]]

                        pal <- palDF[, 
                        "color"
                        ]

                        names
                        (pal) <- palDF[, 
                        "name"
                        ]

                        pal[
                        "-1"
                        ] = 
                        "transparent"

                        plot
                        (fit$points, 
                        col = 
                        pal[
                        primaryClusterNamed
                        (ceObj)], 
                        main = 
                        ""
                        , 
                        pch = 
                        20
                        ,
      
                        xlab = 
                        "Component1"
                        , 
                        ylab = 
                        "Component2"
                        )

                        legend
                        (
                        x = 
                        "topleft"
                        , 
                        legend = names
                        (pal), 
                        cex = 
                        .
                        5
                        ,
	
                        fill = 
                        pal, 
                        title = 
                        "Sample"
                        )
                    


### Cell lineage and pseudotime inference: Slingshot

We now demonstrate how to use the R software package slingshot to infer branching cell lineages and order cells by developmental progression along each lineage. The method, proposed in Street *et al.* (2017), comprises two main steps: (1) The inference of the global lineage structure (i.e., the number of lineages and where they branch) using a minimum spanning tree (MST) on the clusters identified above by RSEC and (2) the inference of cell pseudotime variables along each lineage using a novel method of simultaneous principal curves. The approach in (1) allows the identification of any number of novel lineages, while also accommodating the use of domain-specific knowledge to supervise parts of the tree (e.g., known terminal states); the approach in (2) yields robust pseudotimes for smooth, branching lineages.

The two steps of the Slingshot algorithm are implemented in the functions getLineages and getCurves, respectively. The first takes as input a low-dimensional representation of the cells and a vector of cluster labels. It fits an MST to the clusters and identifies lineages as paths through this tree. The output of getLineages is an object of class SlingshotDataSet containing all the information used to fit the tree and identify lineages. The function getCurves then takes this object as input and fits simultaneous principal curves to the identified lineages. These functions can be run separately, as below, or jointly by the wrapper function slingshot.

From the original published work, we know that the start cluster should correspond to HBCs and the end clusters to MV, mOSN, and mSUS cells. Additionally, we know that GBCs should be at a junction before the differentiation between MV and mOSN cells ( Figure 2). The correspondance between the clusters we found here and the original clusters is as follows.



                        table
                        (
                        data.frame
                        (
                        original = 
                        publishedClusters, 
                        ours = primaryClusterNamed
                        (ceObj)))
                    




                        ## 	   ours
## original -1 c1 c2 c3 c4 c5 c6 c7
## 	 -2 49 40  6 35 11  5  3  2
## 	 1  40 50  0  0  0  0  0  0
## 	 2   1  0 24  0  0  0  0  0
## 	 3   2  2 49  1  0  0  0  0
## 	 4   4  1  0 30  0  0  0  0
## 	 5  36 54  0  3  0  0  0  0
## 	 7   5  0  0 53  0  0  0  0
## 	 8  27  0  0  0  0  0  0  0
## 	 9   3  0  1  1  0 67  2  0
## 	 10  1  0  0  0  0  0 25  0
## 	 11  2  2 17  0  0  0  0  0
## 	 12  0  0  0  0  0 35  0  0
## 	 14  5  0  1  0  0  0 20  0
## 	 15  0  0  1  0  0  0  0 31
                    


**Table:** 

Cluster name	Description	Color	Correspondence
c1	HBC	blue	original 1, 5
c2	GBC	green	original 2, 3, 11
c3	mSUS	red	original 4, 7
c4	Contaminants	orange	original -2
c5	mOSN	purple	original 9, 12
c6	Immature Neurons	brown	original 10, 14
c7	MV	cyan	original 15

**Figure 14.**
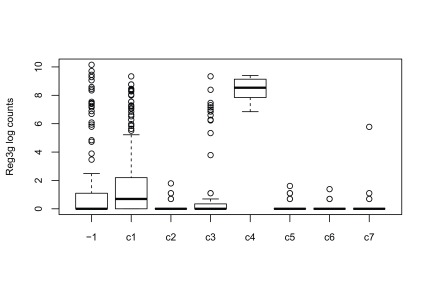
RSEC: Boxplots of the log count of gene Reg3g stratified by cluster.



Cells in cluster c4 have a cluster label of -2 in the original published clustering, meaning that they were not assigned to any cluster. These cells were actually identified as non-sensory contaminants, as they overexpress gene Reg3g (see Figure S1 from Fletcher *et al.* (2017) and Figure 14), and were removed from the original published clustering. While it is reassuring that our workflow clustered these cells separately, with no influence on the clustering of the other cells, we removed cluster c4 to infer lineages and pseudotimes, as cells in this cluster do not participate in the cell differentiation process. Note that, out of the 77 cells overexpressing Reg3g, 11 are captured in cluster c4 and 21 are unclustered in our workflow’s clustering (see Figure 14). However, we retain the remaining 45 cells to infer lineages as they did not seem to influence the clustering.

                        c4 <- 
                        rep
                        (
                        "other clusters"
                        , 
                        ncol
                        (se))

                        c4[
                        primaryClusterNamed
                        (ceObj) == 
                        "c4"
                        ] <- 
                        "cluster c4"

                        boxplot
                        (
                        log1p
                        (
                        assay
                        (se)[
                        "Reg3g"
                        , ]) ~ 
                        primaryClusterNamed
                        (ceObj),
         
                        ylab = 
                        "Reg3g log counts"
                        , 
                        cex.axis = 
                        .
                        8
                        , 
                        cex.lab = 
                        .
                        8
                        )
                    


To infer lineages and pseudotimes, we apply Slingshot to the 4-dimensional MDS of the low-dimensional matrix W. We found that the Slingshot results were robust to the number of dimensions *k* for the MDS (we tried *k* from 2 to 5). Here, we use the unsupervised version of Slingshot, where we only provide the identity of the start cluster but not of the end clusters.



                        our_cl <- 
                        primaryClusterNamed
                        (ceObj)

                        cl <- our_cl[!our_cl %in% c
                        (
                        "-1"
                        , 
                        "c4"
                        )]

                        pal <- pal[!
                        names
                        (pal) %in% 
                        c
                        (
                        "-1"
                        , 
                        "c4"
                        )]

                        X <- W[!our_cl %in% 
                        c
                        (
                        "-1"
                        , 
                        "c4"
                        ), ]

                        mds <- 
                        cmdscale
                        (
                        dist
                        (X), 
                        eig = 
                        TRUE
                        , 
                        k = 
                        4
                        )

                        X <- mds$points


                        lineages <- 
                        getLineages
                        (X, 
                        clusterLabels = 
                        cl, 
                        start.clus = 
                        "c1"
                        )
                    


**Figure 15.**
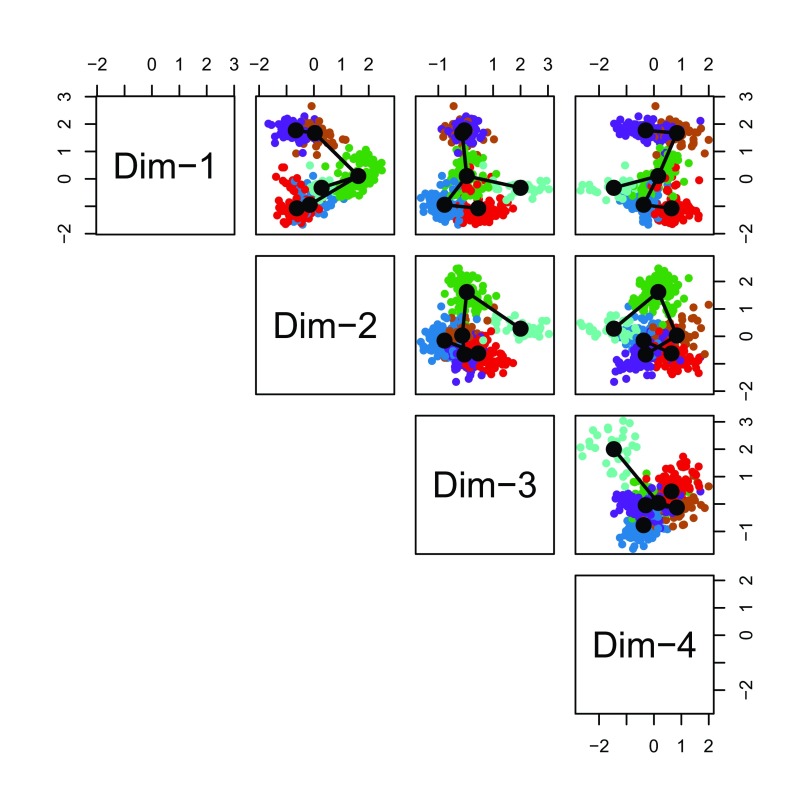
Slingshot: Cells color-coded by cluster in a 4-dimensional MDS space, with connecting lines between cluster centers representing the inferred global lineage structure.



Before fitting the simultaneous principal curves, we examine the global structure of the lineages by plotting the MST on the clusters. This shows that our implementation has recovered the lineages found in the published work ( Figure 15). The slingshot package also includes functionality for 3-dimensional visualization as in Figure 2, using the plot3d function from the package rgl.

                        pairs
                        (lineages, 
                        type=
                        "lineages"
                        , 
                        col = 
                        pal[cl])
                    


**Figure 16.**
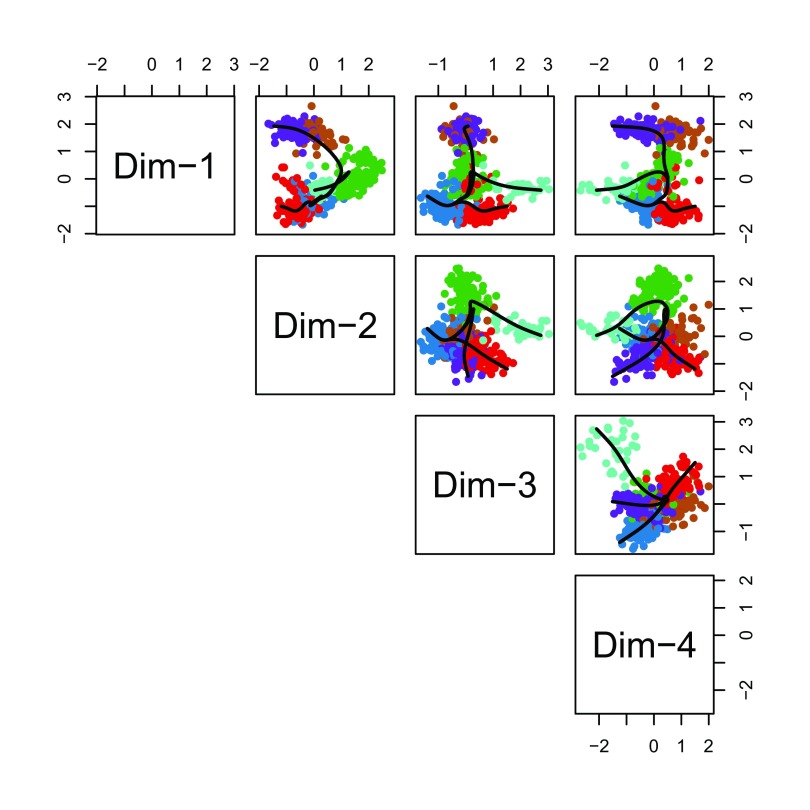
Slingshot: Cells color-coded by cluster in a 4-dimensional MDS space, with smooth curves representing each inferred lineage.



Having found the global lineage structure, we now construct a set of smooth, branching curves in order to infer the pseudotime variables. Simultaneous principal curves are constructed from the individual cells along each lineage, rather than the cell clusters. This makes them more stable and better suited for assigning cells to lineages. The final curves are shown in Figure 16.

                        lineages <- 
                        getCurves
                        (lineages)

                        pairs
                        (lineages, 
                        type=
                        "curves"
                        , 
                        col = 
                        pal[cl])


                        lineages

## class: SlingshotDataSet
##
##  Samples Dimensions
##      561          4
##
## lineages: 3
## Lineage1: c1  c2  c6  c5
## Lineage2: c1  c2  c7
## Lineage3: c1  c3
##
## curves: 3
## Curve1: Length: 7.7816  Samples: 362.44
## Curve2: Length: 7.6818  Samples: 272.31
## Curve3: Length: 4.5271  Samples: 266.81
                    


In the workflow, we recover a reasonable ordering of the clusters using the unsupervised version of slingshot. However, in some other cases, we have noticed that we need to give more guidance to the algorithm to find the correct ordering. getLineages has the option for the user to provide known end cluster(s). Here is the code to use slingshot in a supervised setting, where we know that clusters c3 and c7 represent terminal cell fates.



                        lineages <- 
                        getLineages
                        (X, 
                        clusterLabels = 
                        cl, 
                        start.clus = 
                        "c1"
                        ,
                            
                        end.clus = c
                        (
                        "c3"
                        , 
                        "c7"
                        ))

                        lineagees <- 
                        getCurves
                        (lineages)

                        pairs
                        (lineages, 
                        type=
                        "curves"
                        , 
                        col = 
                        pal[
                        primaryClusterNamed
                        (ceObj)])

                        pairs
                        (lineages, 
                        type=
                        "lineages", 
                        col = 
                        pal[
                        primaryClusterNamed
                        (ceObj)],
       
                        show.constraints = 
                        TRUE
                        )


                        lineages
                    


### Differential expression analysis along lineages

After assigning the cells to lineages and ordering them within lineages, we are interested in finding genes that have non-constant expression patterns over pseudotime.

**Figure 17.**
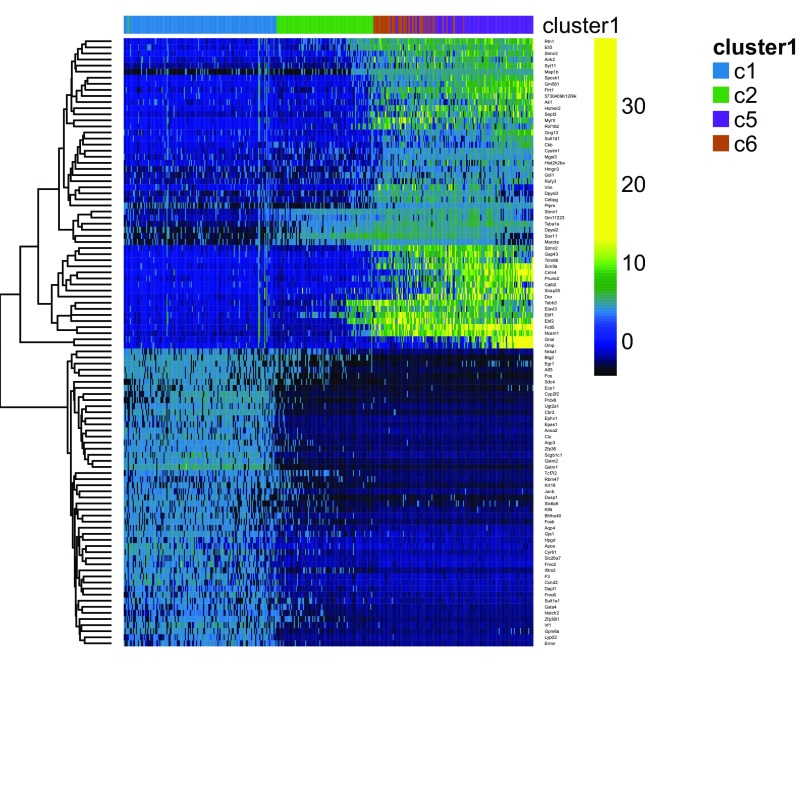
DE: Heatmap of the normalized expression measures for the 100 most significantly DE genes for the neuronal lineage, where rows correspond to genes and columns to cells ordered by pseudotime.



More formally, for each lineage, we use the robust local regression method loess to model in a flexible, non-linear manner the relationship between a gene’s normalized expression measures and pseudotime. We then can test the null hypothesis of no change over time for each gene using the gam package. We implement this approach for the neuronal lineage and display the expression measures of the top 100 genes by p-value in the heatmap of Figure 17.

                        t <- 
                        pseudotime
                        (lineages)[,
                        1
                        ]

                        y <- 
                        assays
                        (se)$normalizedValues[, !our_cl %in% 
                        c
                        (
                        "-1"
                        , 
                        "c4"
                        )]

                        gam.pval <- 
                        apply
                        (y,
                        1
                        ,function(z){
  
                        d <- 
                        data.frame(
                        z=
                        z, 
                        t=
                        
                            t)
                        
  
                        tmp <- 
                        gam
                        (z ~ 
                        lo
                        (t), 
                        data=
                        d)
  
                        p <- 
                        summary
                        (tmp)[
                        4
                        ][[
                        1
                        ]][
                        1
                        ,
                        5
                        ]
  
                        p
})
                    




                        topgenes <- 
                        names
                        (
                        sort
                        (gam.pval, 
                        decreasing = 
                        FALSE
                        ))[
                        1
                        :
                        100
                        ]

                        heatdata <- y[
                        rownames
                        (se) %in% 
                        topgenes, 
                        order
                        (t, 
                        na.last = 
                        NA
                        )]

                        heatclus <- cl[
                        order
                        (t, 
                        na.last = 
                        NA
                        )]

                        ce <- 
                        clusterExperiment
                        (heatdata, heatclus, 
                        transformation = 
                        identity)


                        #match to existing colors

                        cols <- 
                        clusterLegend
                        (ceObj)$combineMany[, 
                        "color"
                        ]

                        names
                        (cols) <- 
                        clusterLegend
                        (ceObj)$combineMany[, 
                        "name"
                        ]

                        clusterLegend
                        (ce)$cluster1[, 
                        "color"
                        ] <- cols[
                        clusterLegend
                        (ce)$cluster1[, 
                        "name"
                        ]]


                        plotHeatmap
                        (ce, 
                        clusterSamplesData = 
                        "orderSamplesValue"
                        , 
                        breaks = 
                        .
                        99
                        )
                    


### Further developments

In an effort to improve scRNA-seq data analysis workflows, we are currently exploring a variety of applications and extensions of our ZINB-WaVE model. In particular, we are developing a method to impute counts for dropouts; the imputed counts could be used in subsequent steps of the workflow, including dimensionality reduction, clustering, and cell lineage inference. In addition, we are extending ZINB-WaVE to identify differentially expressed genes, both in terms of the negative binomial mean and the zero inflation probability, reflecting, respectively, gradual DE and on/off DE patterns. We are also developing a method to identify genes that are DE either within or between lineages inferred from Slingshot.

Finally, a new S4 class called SingleCellExperiment is currently under development ( https://github.com/drisso/SingleCellExperiment). This new class is essentially a SummarizedExperiment class with a couple of additional slots, the most important of which is reducedDims, which, much like the assays slot of SummarizedExperiment, can contain one or more matrices of reduced dimension. This new SingleCellExperiment class would be a valuable addition to the workflow, as we could store in a single object the raw counts as well as the low-dimensional matrix created by the ZINB-WaVE dimensionality reduction step. Once the implementation of this class is stable, we would like to incorporate it to the workflow.

## Conclusion

This workflow provides a tutorial for the analysis of scRNA-seq data in R/Bioconductor. It covers four main steps: (1) dimensionality reduction accounting for zero inflation and over-dispersion and adjusting for gene and cell-level covariates; (2) robust and stable cell clustering using resampling-based sequential ensemble clustering; (3) inference of cell lineages and ordering of the cells by developmental progression along lineages; and (4) DE analysis along lineages. The workflow is general and flexible, allowing the user to substitute the statistical method used in each step by a different method. We hope our proposed workflow will ease technical aspects of scRNA-seq data analysis and help with the discovery of novel biological insights.

## Software and data availability

The source code for this workflow can be found at https://github.com/fperraudeau/singlecellworkflow. Archived source code as at time of publication: http://doi.org/10.5281/zenodo.826211 ( Perraudeau *et al.*, 2017).

The four packages used in the workflow ( scone, zinbwave, clusterExperiment, and slingshot) are Bioconductor R packages and are available at, respectively, https://bioconductor.org/packages/scone, https://bioconductor.org/packages/zinbwave, https://bioconductor.org/packages/clusterExperiment, and https://github.com/kstreet13/slingshot.

Data used in this workflow are available from NCBI GEO, accession GSE95601.

## Supplementary material

Supplementary File 1: **sessionInfo**.
