# Supplementary material for: Bioconductor workflow for single-cell RNA sequencing: Normalization, dimensionality reduction, clustering, and lineage inference
Source: F1000Res. 2017 Jul 21;6:1158. [Version 1] doi: 10.12688/f1000research.12122.1 (PMC5558107; doi:10.12688/f1000research.12122.1)
Supplement: Supplementary file 1 [file f1000research-6-13120-s0000.tgz › 254521cd-3372-444e-96cb-4efd0d7a44d2.pdf]

```
sessionInfo ()
## R version 3.4.0 (2017-04-21)
## Platform: x86_64-apple-darwin15.6.0 (64-bit)
## Running under: macOS Sierra 10.12.5
##
## Matrix products: default
## BLAS: /Library/Frameworks/R.framework/Versions/3.4/Resources/lib/libRblas.0.dylib
## LAPACK: /Library/Frameworks/R.framework/Versions/3.4/Resources/lib/libRlapack.dylib
##
## locale:
## [1] en_US.UTF-8/en_US.UTF-8/en_US.UTF-8/C/en_US.UTF-8/en_US.UTF-8
##
## attached base packages:
##  [1] splines   stats4    parallel  stats     graphics  grDevices utils
##  [8] datasets  methods   base
##
## other attached packages:
##  [1] RColorBrewer_1.1-2         gam_1.14-4
##  [3] doParallel_1.0.10          iterators_1.0.8
##  [5] foreach_1.4.3              slingshot_0.1.1
##  [7] princurve_1.1-12           zinbwave_0.99.6
##  [9] scone_1.1.2                clusterExperiment_1.3.2
## [11] SummarizedExperiment_1.6.3 DelayedArray_0.2.7
## [13] matrixStats_0.52.2         Biobase_2.36.2
## [15] GenomicRanges_1.28.3       GenomeInfoDb_1.12.2
## [17] IRanges_2.10.2             S4Vectors_0.14.3
## [19] BiocGenerics_0.22.0        BiocParallel_1.10.1
## [21] knitr_1.16                 BiocStyle_2.4.0
##
## loaded via a namespace (and not attached):
##   [1] copula_0.999-17          uuid_0.1-2
```

```
##   [3] backports_1.1.0        aroma.light_3.6.0
##   [5] NMF_0.20.6             igraph_1.0.1
##   [7] plyr_1.8.4             lazyeval_0.2.0
##   [9] pspline_1.0-18         rncl_0.8.2
##  [11] ggplot2_2.2.1          gridBase_0.4-7
##  [13] digest_0.6.12          htmltools_0.3.6
##  [15] viridis_0.4.0          gdata_2.18.0
##  [17] magrittr_1.5           memoise_1.1.0
##  [19] cluster_2.0.6          mixtools_1.1.0
##  [21] limma_3.32.2           Biostrings_2.44.1
##  [23] annotate_1.54.0        bayesm_3.1-0
##  [25] stabledist_0.7-1       rARPACK_0.11-0
##  [27] R.utils_2.5.0          prettyunits_1.0.2
##  [29] colorspace_1.3-2       blob_1.1.0
##  [31] BiocWorkflowTools_1.2.0  dplyr_0.7.1
##  [33] hexbin_1.27.1          RCurl_1.95-4.8
##  [35] jsonlite_1.5           genefilter_1.58.1
##  [37] bindr_0.1              phylobase_0.8.4
##  [39] survival_2.41-3        ape_4.1
##  [41] glue_1.1.1             registry_0.3
##  [43] gtable_0.2.0           zlibbioc_1.22.0
##  [45] XVector_0.16.0         compositions_1.40-1
##  [47] kernlab_0.9-25         prabclus_2.2-6
##  [49] DEoptimR_1.0-8         scales_0.4.1
##  [51] DESeq_1.28.0           mvtnorm_1.0-6
##  [53] edgeR_3.19.3           DBI_0.7
##  [55] rngtools_1.2.4         Rcpp_0.12.11
##  [57] viridisLite_0.2.0      xtable_1.8-2
##  [59] progress_1.1.2         bit_1.1-12
##  [61] bold_0.4.0             mclust_5.3
##  [63] glmnet_2.0-10          htmlwidgets_0.8
```

```
##  [65] httr_1.2.1             gplots_3.0.1
##  [67] fpc_2.1-10             modeltools_0.2-21
##  [69] pkgconfig_2.0.1        reshape_0.8.6
##  [71] XML_3.98-1.9           R.methodsS3_1.7.1
##  [73] flexmix_2.3-14         nnet_7.3-12
##  [75] locfit_1.5-9.1         softImpute_1.4
##  [77] howmany_0.3-1          rlang_0.1.1
##  [79] reshape2_1.4.2         AnnotationDbi_1.38.1
##  [81] munsell_0.4.3          tools_3.4.0
##  [83] RSQLite_2.0            ade4_1.7-6
##  [85] evaluate_0.10.1        stringr_1.2.0
##  [87] yaml_2.1.14            bit64_0.9-7
##  [89] robustbase_0.92-7      rgl_0.98.1
##  [91] caTools_1.17.1         dendextend_1.5.2
##  [93] bindrcpp_0.2           EDASeq_2.10.0
##  [95] nlme_3.1-131           mime_0.5
##  [97] whisker_0.3-2          taxize_0.8.8
##  [99] R.oo_1.21.0            xml2_1.1.1
## [101] biomaRt_2.32.1         compiler_3.4.0
## [103] tibble_1.3.3           geneplotter_1.54.0
## [105] pcaPP_1.9-72           gsl_1.9-10.3
## [107] RNeXML_2.0.7           stringi_1.1.5
## [109] GenomicFeatures_1.28.4 RSpectra_0.12-0
## [111] lattice_0.20-35        trimcluster_0.1-2
## [113] Matrix_1.2-10          tensorA_0.36
## [115] ADGofTest_0.3          data.table_1.10.4
## [117] bitops_1.0-6           httpuv_1.3.5
## [119] rtracklayer_1.36.3     R6_2.2.2
## [121] latticeExtra_0.6-28    hwriter_1.3.2
## [123] bookdown_0.4           ShortRead_1.34.0
## [125] KernSmooth_2.23-15     gridExtra_2.2.1
```

```
## [127] codetools_0.2-15        energy_1.7-0
## [129] boot_1.3-19             MASS_7.3-47
## [131] gtools_3.5.0            assertthat_0.2.0
## [133] rhdf5_2.20.0            pkgmaker_0.22
## [135] rprojroot_1.2           RUVSeq_1.10.0
## [137] GenomicAlignments_1.12.1 Rsamtools_1.28.0
## [139] GenomeInfoDbData_0.99.0  locfdr_1.1-8
## [141] diptest_0.75-7          grid_3.4.0
## [143] tidyr_0.6.3             class_7.3-14
## [145] rmarkdown_1.6           segmented_0.5-2.1
## [147] shiny_1.0.3             numDeriv_2016.8-1
```
