# Supplementary material for: Bioconductor workflow for single-cell RNA sequencing: Normalization, dimensionality reduction, clustering, and lineage inference
Source: F1000Res. 2017 Jul 21;6:1158. [Version 1] doi: 10.12688/f1000research.12122.1 (PMC5558107; doi:10.12688/f1000research.12122.1)
Supplement: Peer review history

# Peer review: Referee response for version 1

**DOI:** 10.5256/f1000research.13120.r24387

**Published:** 2017-08-07

**Authors:** Michael I. Love

**Affiliations:** 1 Department of Biostatistics, Department of Genetics, University of North Carolina at Chapel Hill, Chapel Hill, NC, USA

The authors have developed an easy to follow workflow which goes beyond preparing single-cell data for analysis, showing how to use existing methods and packages to normalize, perform dimension reduction, construct cell lineages and perform differential testing along those lineages. The workflow seems like it will be useful, and I hope the authors can update the workflow as new frameworks come into play (e.g. SingleCellExperiment).

 I have the following suggestions for improving the workflow:

 It would be useful to put a link to the source code (or to the section where the link to the source code exists) near the top of the document.

 I was confused a bit by "the first major bifurcation in the HBC lineage trajectory occurs prior to cell division". Can you be more specific about what you are referring to here by cell division, as without knowledge of the system, I'm not sure where the cell division you refer to should appear.

 "within a single object": It may be good to explain what an "object" here is. You could, for example, refer to Figure 2 of the Bioconductor Nature Methods paper ^1^.

 Misspelling: "reasonnable"

 On filtering for most variable genes, I understand this decision, and I also recommend it during workshops before making ordination plots. I know that students are not always certain why we care about variance (unsupervised). I like to mention that these are the genes where the "action" is. A side point, the log(x+1) is not variance stabilizing for RNA-seq counts in general. This filter can give higher priority to low count genes than to genes where there is interesting biological variability (though I do not doubt that the very high biological variance genes will be preserved). It might be useful to show a vsn::meanSdPlot() for the matrix log1p(assay(se))? 

 "correcting for batch effects": What are batch effects? (Of course, I know what they are, but a reader may not, and you could cite some of the single cell literature here.)

 "Note that, in this case, the low-dimensional matrix W is not included in the computation of residuals to avoid the removal of the biological signal of interest.": I understood this sentence only on a second pass through. One problem is that you haven't defined W in the text yet (only in Figure 4). I would only reference the matrix W if you have defined it.

 Figure 6: Can you change the figure width so that PC1 is not squished?

 Is there a circularity to the recovery of published clusters in Figure 6? Was ZINB-WaVE used in Fletcher (2017)?

 Can you say what the meaning of the color white is in Figure 8 (in the text or caption near this figure)?

 Figure 15 refers back to Figure 2 but does not use the same color scheme for the known cell types, so the reader cannot verify if you've recovered the lineages from the publication. It would be good therefore to have a legend for these figures (Fig 15 and following) indicating which cell types the colors refer to (this information is in the unlabeled table above, but should be included as a legend here).

 Can you briefly describe what a GAM is ahead of Figure 17?

I have read this submission. I believe that I have an appropriate level of expertise to confirm that it is of an acceptable scientific standard.

## References

[1] HuberWCareyVJGentlemanRAndersSCarlsonMCarvalhoBSBravoHCDavisSGattoLGirkeTGottardoRHahneFHansenKDIrizarryRALawrenceMLoveMIMacDonaldJObenchainVOleśAKPagèsHReyesAShannonPSmythGKTenenbaumDWaldronLMorganM: Orchestrating high-throughput genomic analysis with Bioconductor. *Nat Methods*.2015;12(2) : 10.1038/nmeth.3252 115-21 10.1038/nmeth.3252 25633503PMC4509590

# Peer review: Referee response for version 1

**DOI:** 10.5256/f1000research.13120.r24388

**Published:** 2017-08-07

**Authors:** Andrew McDavid

**Affiliations:** 1 Department of Biostatistics, University of Rochester Medical Center, Rochester, NY, USA

Major comments:

1. There is something wrong with the data download link in the F1000 version so that I am unable to download these files and actually reproduce the workflow. I experimented a bit to see if I could figure out how to download the data anyways, but will reserve further evaluation of this submission until this issue can be resolved by the authors.
  ```{r}
  urls = c("https://www.ncbi.nlm.nih.gov/geo/download/?acc=GSE95601&format=file&file=GSE95601%5FoeHBCdiff% "https://raw.githubusercontent.com/rufletch/p63-HBC-diff/master/ref/oeHBCdiff_clusterLabels.)
  ```
2. a. This workflow will likely be out-of-date when the underlying packages transition to use SingleCellExperiment. This is actually a positive thing because many of the more opaque lines of code (involving subsetting ERCC genes, etc) will be more streamlined.
  b. It requires installation of the development branch of bioconductor, which impacts the usefulness of the workflow to the average user. I expect the authors will revise this tutorial when Bioconductor 3.6 is released and use of the devel branch is no longer necessary. Additionally `slingshot` is an requirement, but currently only exists on github and no SHA1 provided. I hope that `slingshot` will be added as a bioconductor package shortly. In the meantime, a tag must be added to the git repo for the release being used in this workflow and instructions provided for how to install this tag. Additionally, the authors may wish to note that installation instructions for the packages will be provided at the end of the workflow so that someone proceeding sequentially will not be tripped up.
  c.Opaque code is presented in order to generate plots, e.g.
  ```{r}
  palDF <- ceObj@clusterLegend[[1]]
  pal <- palDF[, "color"]
  names(pal) <- palDF[, "name"]
  pal["-1"] = "transparent"
  plot(fit$points, col = pal[primaryClusterNamed(ceObj)], main = "", pch = 20, xlab = "Component1", ylab = "Component2") legend(x = "topleft", legend = names(pal), cex = .5, fill = pal, title = "Sample")
  ```
  While this complexity may be necessary, perhaps some of it could be encapsulated as accessor functions in the package? Too much complexity here may cause users to miss the forest for the trees.  
3. The authors could better motivate (or at least explain the impact of) some of the default parameters and procedures.

- Why do we set a zcut threshold of 3 for the `scone` filtering? 
- Why K=50 for zinbwave?
- RSEC parameters
  How should a user decide on a value for these parameters?

I have read this submission. I believe that I have an appropriate level of expertise to confirm that it is of an acceptable scientific standard, however I have significant reservations, as outlined above.

# Peer review: Referee response for version 1

**DOI:** 10.5256/f1000research.13120.r24385

**Published:** 2017-08-03

**Authors:** Stephanie C. Hicks

**Affiliations:** 1 Department of Biostatistics and Computational Biology, Dana–Farber Cancer Institute, Boston, MA, USA

In this article, the authors Perraudeau, Risso, Street, Purdom and Dudoit present a nice workflow for normalization, dimensionality reduction, clustering, and lineage inference of single-cell RNA-seq data (scRNA-seq) using R packages from the open-source Bioconductor project. I enthusiastically agree with the authors on an “increasing need for workflows that integrate these tools to yield a seamless scRNA-seq data analysis pipeline” and this workflow is a great step in the right direction. However, I have some constructive suggestions that will better integrate other previously developed work and improve this workflow.

1. In this workflow, the authors start with a count table. However, the majority of researchers will start with raw reads (e.g. a FASTQ file). It would be great if the author discussed current best practices for the quantification step of scRNA-seq data. Alternatively, the authors could point to other references that have already been developed.
2. I would like to see the authors take advantage of the rich functionality and data exploration tools for cell- and gene-specific quality control (QC) introduced in low-level analysis workflows such as the one from Lun et al. (2016) ^1^. Also, in this workflow, the authors create multiple SummarizedExperiment objects (e.g. one with only the top 1000 highly variable genes (HVGs), one with all genes, etc). This doesn’t seem efficient, especially with large single cell data sets such as the 1.3 million cells from embryonic mouse brains. I think both of these concerns can now be addressed with efforts such as the recently developed SingleCellExperiment Bioconductor object ( https://github.com/drisso/SingleCellExperiment). For example, the authors could add a “USE” column in the gene- or cell-specific meta table to represent whether or not a particular gene in a particular cell met the filtering criteria applied. The authors could store W in the reduceDim assay of the SingleCellExperiment object.
3. In ZINB-WaVE, the authors specify the number of dimensions for the low-dimensional space (K) to be K=50. Could the authors add more details for the reader explaining why they picked K=50 and describe situations in which a user would want to specify a higher or lower K? In particular, it would be useful to discuss computational time in terms of number of genes and cells. Also, it would be useful to note that if you only wanted to use ZINB-WaVE to remove known covariates for normalization, you can use K=0. 

Minor comments:

1. When selecting the top 1000 HVGs, why do the authors not take into account the overall mean-variance relationship and only select genes based on the variance?
2. It would be great if the authors referenced other tools available for similar analyses currently available. For example there are several available packages for normalization of scRNA-seq data, such as calculating global scaling factors can be done with scran ( https://bioconductor.org/packages/release/bioc/html/scran.html) or gene and cell-specific scaling factors using SCnorm ( https://github.com/rhondabacher/SCnorm). Alternatively, users might want to try using relative transcript counts using Census ( https://bioconductor.org/packages/release/bioc/html/monocle.html).

I have read this submission. I believe that I have an appropriate level of expertise to confirm that it is of an acceptable scientific standard, however I have significant reservations, as outlined above.

## References

[1] LunATMcCarthyDJMarioniJC: A step-by-step workflow for low-level analysis of single-cell RNA-seq data with Bioconductor. *F1000Res*.2016;5: 10.12688/f1000research.9501.2 2122 10.12688/f1000research.9501.2 27909575PMC5112579
